# Chinese lantern in *Physalis* is an advantageous morphological novelty and improves plant fitness

**DOI:** 10.1038/s41598-018-36436-7

**Published:** 2019-01-24

**Authors:** Jing Li, Chunjing Song, Chaoying He

**Affiliations:** 1 State Key Laboratory of Systematic and Evolutionary Botany, Institute of Botany, Chinese Academy of Sciences, Nanxincun 20, Xiangshan, 100093 Beijing, China; 2 University of Chinese Academy of Sciences, Yuquan Road 19, 100049 Beijing, China

## Abstract

The origin of morphological novelties is an important but neglected issue of evolutionary biology. The fruit of the genus *Physalis*, a berry, is encapsulated by a novel morphological feature of the post-floral, accrescent calyx that is referred to as a Chinese lantern. The evolutionary developmental genetics of the Chinese lantern have been investigated in the last decade; however, the selective values of the morphological novelty remain elusive. Here, we measured the photosynthetic parameters of the fruiting calyces, monitored microclimatic variation within the Chinese lanterns during fruit development, performed floral-calyx-removal experiments, and recorded the fitness-related traits in *Physalis floridana*. Ultimately, we show that the green-fruiting calyx of *Physalis* has photosynthetic capabilities, thus serving as an energy source for fruit development. Moreover, the developing Chinese lantern provides a microclimate that benefits the development and maturation of berry and seed, and it improves plant fitness in terms of fruit/seed weight and number, and fruit maturation under low-temperature environments. Furthermore, the lantern structure facilitates the dispersal of fruits and seeds by water and wind. Our results suggest that the Chinese lantern morphology of *Physalis* is an evolutionary adaptive trait and improves plant fitness, thus providing new insight into the origin of morphological novelties.

## Introduction

Morphological variations and morphological novelties are assumed to be a result of adaptive evolution. The developmental mechanisms for evolution are summarized for morphological diversification^1–4^, and for the origin of plant morphological novelties^5^. Flowers are a characteristic of angiosperms, and the evolution of the flower form is largely related to facilitating pollination to increase the rate of reproduction^6^. The perianth (sepals and petals) is generally considered to be a protective structure for the central reproductive organs and an attractant for pollinators^7^. In some plants the perianth persists and continues to grow in order to enhance the attraction to pollinators, such as the sepals of the genus *Helleborus*, which are enlarged and as brightly colored as petals^8^. Nonetheless, persistent sepals (calyx), e.g. in Solanaceae, are not necessarily modified for this purpose. The post-floral calyx morphology, including size and shape, at the fruiting stages in Solanaceae is extremely diversified^5,9,10^. In *Physalis*, the post-floral calyx becomes inflated and resembles a Chinese lantern. This structure is also called inflated calyx syndrome (ICS) and this morphological novelty distinguishes *Physalis* from most other genera within the Solanaceae^11^.

How did the novelty arise? We previously found that the origin of the Chinese lantern is associated with the heterotopic expression of the *Physalis floridana* MADS-box gene 2 (*MPF2*) in floral organs^11^. Compared to its ortholog in *Solanum tuberosum*, the heterotopic expression of *MPF2* in the floral organs of *Physalis* may result from variation in the CArG-boxes in its promoter^11^. The phenotypic variation of *MPF2* knockdowns supports the role of *MPF2* in male fertility and the post-fruiting growth of the calyx in *P. floridana*^11^, which was further justified by overexpression of this gene synthesized ICS-like trait in *Solanum*^12^. The Chinese lantern is derived from the floral calyx after pollination without changing organ identity because downregulation of another *P. floridana* MADS-box gene 3 (*MPF3*), which is an euAP1 gene, generates a leaf-like floral calyx, a small lantern, and poor male fertility^13^. Moreover, MPF3 interacts with MPF2 to bind the variants of the CArG-boxes in the *MPF2* promoter^13^, thus governing the floral expression of *MPF2*, which is the key to the origin of the Chinese lantern in *Physalis*.

**Figure 1:**
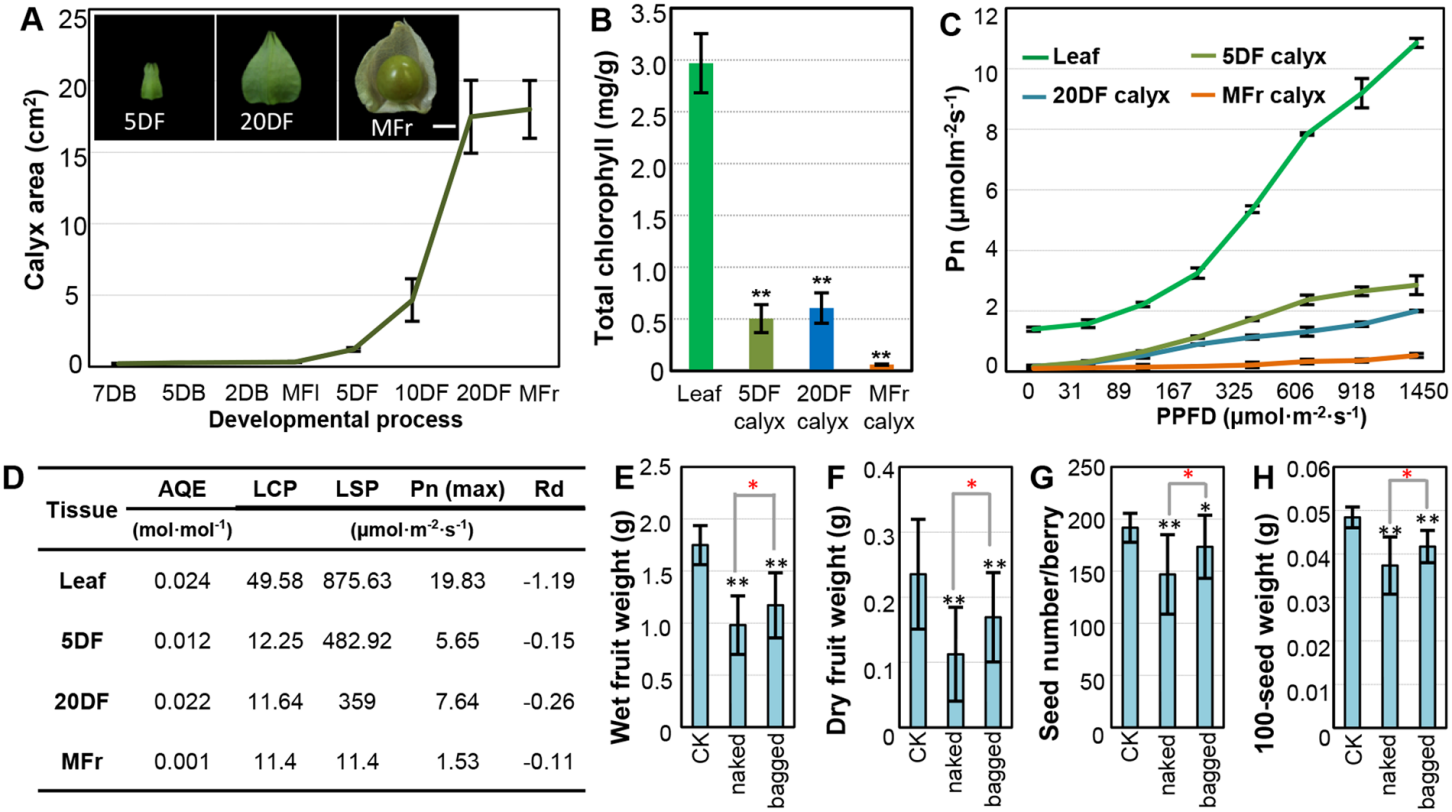
The photosynthetic capability of green calyx contributes to fruit and seed development in *Physalis floridana*. (**A**) Quantification of calyx areas at different developmental stages in *P. floridana*. The blooming flower (MFl) stage was set as 0 day, and then floral buds were defined as 2, 5, and 7 days before flower open (DB), and fruit stages include the developing fruits 5, 10, and 20 days after fertilization (DF) and mature fruits (MFr). Bar = 5 mm. (**B**) Total chlorophyll content in the indicated tissues. (**C**) Light response curve of the indicated tissues. Pn, net photosynthetic rate; PPFD, photosynthetic photon flux density. (**D**) The photosynthetic parameters. AQE, apparent quantum efficiency; LCP, light compensation point; LSP, light saturation point; Pn (max), the max net photosynthetic rate; Rd, dark respiration rate. These parameters reflect photosynthetic efficiency in different green tissues as indicated. (**E**,**F**) Wet and dry fruit weight under different conditions. CK, the intact *Physalis* fruits; naked, the naked *Physalis* berries; bagged, the bagged *Physalis* berries after removing the calyx. (**G**,**H**) Seed number per berry and 100-seed weight from different fruits as indicated. The significance of difference is indicated by ^*^(*P* ≤ 0.05) and ^**^(*P* ≤ 0.01). The black stars indicate the difference significance relative to CK, whereas the red stars indicate the difference between the naked and the bagged berries.

The Chinese lantern-like structure is found in a few additional genera of the Solanaceae, such as *Margaranthus*, *Przewalskia*, and *Withania*^10^, which evidence for its phylogenetic polyphyly^14,15^; however, the expression of *MPF2*-like genes in floral organs is plesiomorphic within the family Solanaceae^10^. Moreover, *MPF2*-like variations (in both floral expression and protein-protein interactions) define the evolutionary pattern of the ICS in Solanaceae^10,16,17^. Furthermore, fossils of lantern-like fruits from the Eocene (52.2-Ma) suggest the structure has an ancient history and that the origin of ICS occurred in a lakeside rainforest paleoenvironment^18^. Therefore, Solanaceae is a logical family with which to study the genetics, biosynthesis, and evolution of ICS-like traits^10–12,16–18^. The selective value of the Chinese lantern remains unknown; its potential adaptive functions have been discussed previously^19^, and include facilitating wind-mediated fruit dispersal^9^, maintaining an adaptive microclimate^16^, originating as a byproduct of fertility evolution^11,20^, and being a source of photosynthesis and protection of inner organs^12^. This accessory structure may benefit berry development and seed dispersal, but empirical evidence is lacking. Thus, this study aimed to assess the potential selective values of the Chinese lantern in *Physalis* by evaluating its biological and ecological roles.

## Results

### *Physalis* calyx photosynthesizes and contributes to berry and seed weight

After fertilization, the calyx of *Physalis floridana* started to inflate and surround the developing berry, as documented by images of 5 days after fertilization (5DF), 20 days after fertilization (20DF), and mature calyces (MFr; Fig. 1A). Because the calyx is green before the berry matures, we hypothesized that it could provide photosynthate for the berry. To verify this, we first measured chlorophyll content as an indicator of photosynthetic activity. We found that the chlorophyll content was low (~0.06 mg/g) in the mature calyx, and it was 0.5 mg/g 5 DF and 0.61 mg/g 20 DF, even lower than the levels in leaf tissues (~2.91 mg/g) (Fig. 1B). This suggested the inflated calyx had potential to photosynthesize. To confirm this, we measured photon flux density response curves, which reflect accumulation of carbohydrate of photosynthesis. Overall, net photosynthetic rate (Pn) of both calyces and leaves shared similar variation to increase with increasing photosynthetic photon flux density (Fig. 1C). However, calyces and leaves varied in their photon response parameters (Fig. 1D), indicating different photosynthetic efficiency and capacity. Unlike in leaves, the apparent quantum efficiency (AQE), the light compensation point (LCP), light saturation point (LSP) difference, and the max net photosynthetic rate [Pn (max)] was low in all calyces. However, the Pn (max) of green calyces 5 DF and 20 DF were overall higher than that of MFr calyces (Fig. 1D). In contrast, dark respiration rate (Rd) was highest in MFr calyces (Fig. 1D). These suggest green calyx has the capability for photosynthesis.

To reveal the benefits of the calyx to the berry, we conducted calyx-removal experiments at the blooming stage. Calyces were completely removed, and the remaining floral organs were left untouched and covered with small waterproof and non-transparent paper bags. These were designated the bagged *Physalis* berries. Intact *Physalis* fruits were included as controls. Artificial crosses standardized the fruit setting rate, seed number per berry, and measures of maturation time. Characters of mature fruits, such as fruit weight (wet and dry), seed number per berry, and 100-seed weight, were recorded. When calyx and carpopodium turned yellow (Fig. S1A,B), the fruit could easily be removed from the mother plant, indicating maturation of the fruit of *P. floridana*. We found, in the bagged berries, these fruit features showed significant decrease compared with those from the intact flowers. Removing the calyx could lead to a 28%–33% reduction in dry and wet berry weight, a 9.4% decrease in seed number per berry, and a 14% loss in 100-seed weight in the bagged *Physalis* fruits (Fig. 1E–G). However, when the remaining floral organs were naked, the weights of the berry and seeds were further decreased. Wet and dry weight of naked berries was decreased by 44% and 40% respectively compared with the intact fruits containing the Chinese lantern (Fig. 1E–F). Seed number per berry declined by about 23% (Fig. 1G), and 100-grain weight was reduced by ~23% compared with the intact fruits (Fig. 1H). The extent of reduction to both wet and dry weight in the naked and bagged experiments was comparable to that of intact *Physalis* fruits (only wet fruits were weighted as fruit or berry weight in the following experiments). Besides the obvious differences between the fruit-character values of naked and bagged *Physalis* berries as demonstrated above, the survival rate of mature fruits showed a similar reduction pattern in these cases compared to the intact one (Fig. S1C). In addition, the intact fruits and the bagged naked berries nearly became mature at the same time, whereas the naked *Physalis* berry matured earlier (Fig. S1D). These observations not only indicate that the green fruiting calyx is a photosynthesis source and contributes to fruit weight and seed development, but also suggest that the Chinese lantern might also act as a regulator of microclimate, which is required for fruit development in *P. floridana* (see below).

### Chinese lanterns serve a microclimate during fruit development

**Figure 2:**
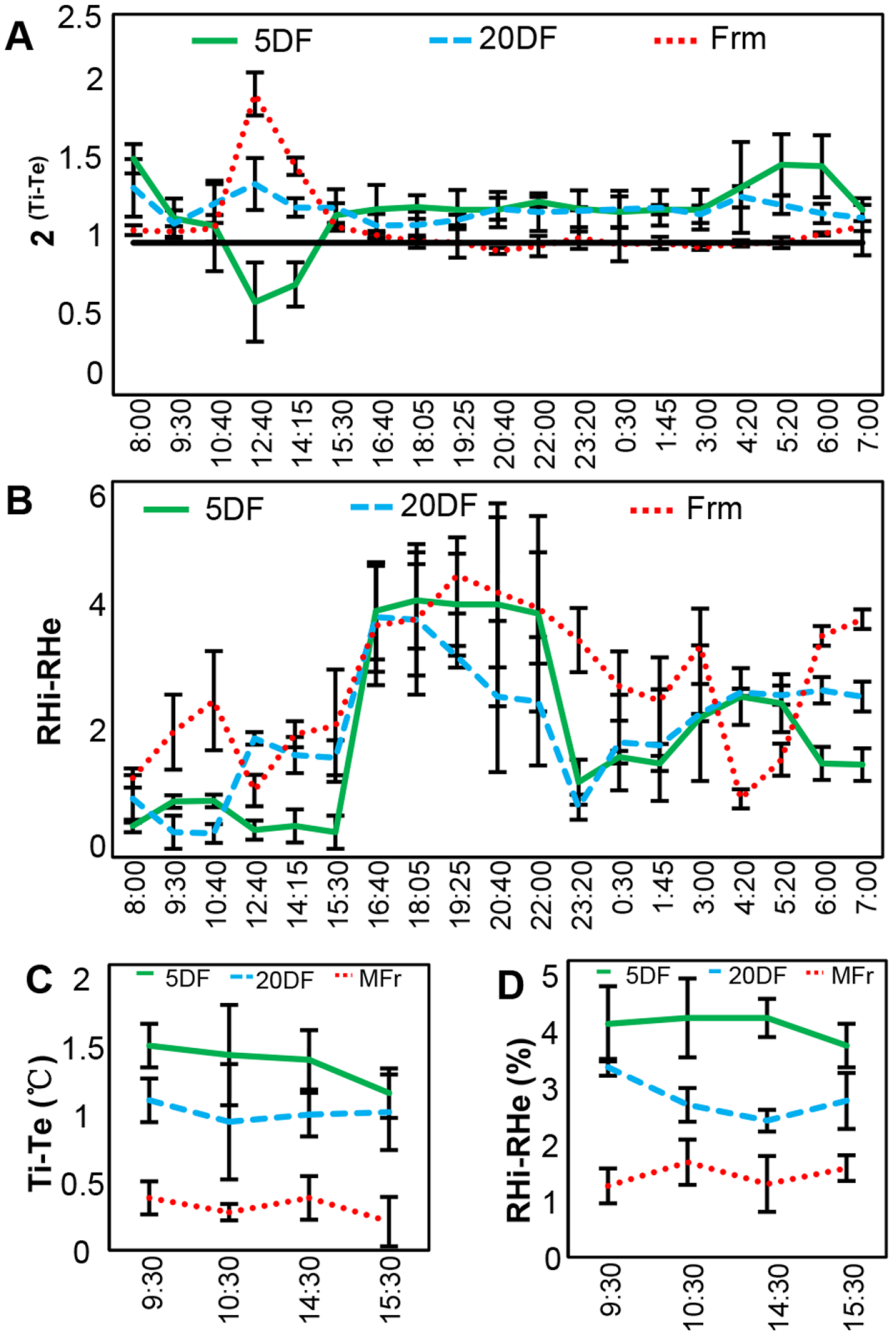
Chinese lantern in *Physalis floridana* maintains a microclimate. (**A**) Diurnal variation of internal and external temperature of the Chinese lantern for three developmental stages as indicated in Fig. 1. Ti and Te indicate internal and external temperature of the lantern, respectively. 2^(Ti-Te)^ was used to indicate the internal and external temperature difference of lanterns. Black horizontal line indicates no Ti and Te difference. (**B**) Variation of internal and external relative humidity (RH) of the Chinese lanterns in three developmental stages. RHi and RHe indicate internal and external RH of the Chinese lanterns, respectively. RHi-RHe indicates the internal and external RH difference of lanterns. (**C**) Variation of internal temperature of the Chinese lantern at three developmental stages under 16 °C conditions. (**D**) Variation of RHi relative to RHe of the Chinese lantern at three developmental stages under 16 °C conditions.

To further confirm the role of the lantern microclimate favorable to fruit development, we measured variation of temperature and relative humidity (RH) within the calyx lantern under various conditions. Under normal growth conditions, diurnal variation of temperature and RH was monitored. Temperature within the lantern (Ti) of three developmental stages (5 DF, 20 DF and MFr) varied differently as external temperature (Te) changed (Fig. 2A). Peak of variation value [2^(Ti-Te)^] of three periods all appeared around 13:00, the hottest time point in a day, and the internal temperature of 5 DF lantern was lower than the external one, which is opposite to that of Chinese lantern stages of MFr and 20 DF (Fig. 2A). Besides the peak around 13:00, the Ti in 5 DF and 20 DF lanterns was always slightly higher than the Te, while the Ti in MFr lantern was almost similar to the Te (Fig. 2A). However, the internal RH (RHi) of Chinese lanterns was higher than the external RH (RHe) in all three developmental stages; however, the extent of the differences were slightly different (Fig. 2B), indicating that Chinese lanterns may keep moisture inside. These results suggest that the Chinese lantern syndrome could serve a microclimate to buffer variations in external temperatures and humidity during fruit development.

We also observed that both Ti and RHi were higher than external ones in 20 DF lanterns as the Te dropped from 28 °C to 10 °C, and as the RHe changed from 55% to 30% (Fig. S2). Furthermore, the Ti in the lantern of the three stages in response to Te variation was different under natural conditions (Fig. 2A), possibly reflecting differences in their capability to buffer when challenged with variations in temperature. To verify this, we simultaneously monitored the Ti and RHi under constant 16 °C conditions, and found that the lanterns of 5 DF, 20 DF and MFr maintained Ti and RHi higher than those outside to different extents (Fig. 2C,D), hinting that the younger lanterns tend to be more efficient at maintaining an internal microclimate than more mature lanterns.

Unlike the intact berries, *Physalis* berries that had the calyx removed became shriveled and wrinkled after one month, and had significantly lower weight (Fig. S3), possibly as a result of water loss. We conclude that the Chinese lantern structure keeps the berry fresh and in a good condition for a long period after maturation by serving as a regulator of microclimate.

### The lantern microclimate enhances fruit development, thus improving fitness

To further confirm the microclimate role of the Chinese lantern in the fruit development, we subjected *Physalis* seedlings at the blooming stage (Fig. S4) to low temperature (16 °C) conditions. First, we evaluated the effect of low temperature on flower and fruit development. Compared to optimal growth conditions, low temperature made the vegetative organs of a flower apparently smaller (Fig. S4A,B), while the reproductive organs seemed unaffected in both morphology and function (Fig. S4D–G). Moreover, the low temperature significantly extended fruit growth-maturation time from 29 to 50 days (Fig. S4H); Furthermore, compared with the fruits developing under normal conditions, fruit setting rate, fruit weight, number of seeds per berry and 100-grain weight were significantly reduced (Fig. S4I–L), thus reducing fitness.

**Figure 3:**
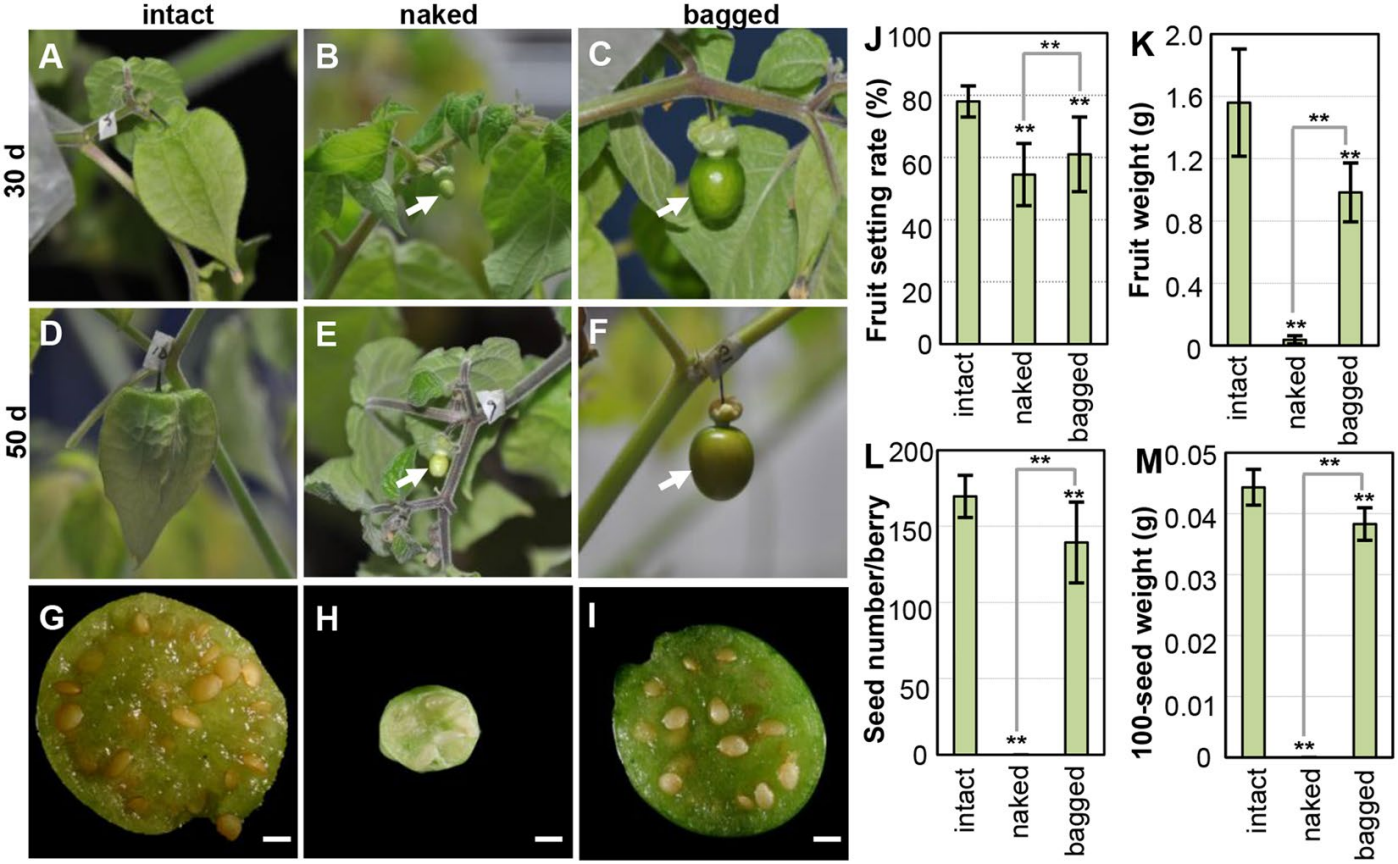
Chinese lantern improves *Physalis* fitness in terms of fruit and seed development under low temperature (16 °C). (**A–C**) Fruits 30 d after fertilization. (**A**) An intact fruit. (**B**) A naked berry. (**C**) A bagged berry; paper bag removed. (**D–F**) Fruits 50 d after fertilization. (**D**) An intact fruit. (**E**) A naked berry. (**F**) A bagged berry; paper bag removed. Arrows indicate berries without a calyx. d, day. (**G**–**I**) Section of mature fruits. (**G**) An intact fruit. (**H**) A naked berry. (**I**) A bagged berry; paper bag removed. Bars = 5 mm. Mature seeds were not produced in the small, naked berry that was grown at 16 °C conditions. **(J–L)** Quantification of fruit setting rate (**J**) fruit weight (**K**) seed number per berry (**L**) and 100-seed weight (**M**) of the berries as indicated. The significance of differences is indicated by ^*^(*P* ≤ 0.05) and ^**^(*P* ≤ 0.01).

We next estimated the potential benefit of the Chinese lantern for fruit development under such a cold environment via calyx-removal experiments. Figure 3A–C showed the representative state of the 30 DF intact fruits, naked berries, and bagged berries of *P. floridana*, which were not mature until 50 DF (Fig. 3D–F). The mature seed number per berry was reduced (Fig. 3G–I); specifically, the naked berry development was arrested (Fig. 3B,E), and mature seeds were hardly developed (Fig. 3H). The fruit setting rate was decreased (Fig. 3J), although artificial crosses were performed. Fruit weight of the berries was significantly reduced to 63% (bagged) and 3% (naked) compared with the intact fruit weight (Fig. 3K). The mature seed number per berry in the bagged berries was also reduced compared with that in the intact fruits (Fig. 3L). Moreover, 100-seed weight was significantly reduced in the bagged berry as a result of removing the lantern (Fig. 3M).

A reduced fitness was also observed in tomato (Micro-Tom) plants that were placed in 16 °C incubator after flowering (Fig. S5). Maturation time differed under the low temperature conditions compared with tomato grown under normal conditions (Fig. S5A–G). Most berries stopped growing and were aborted under the low temperature conditions (Fig. S5B,E). Some fruits in the bagged and naked treatments developed to maturity, but both fruit size and seed number per berry were reduced (Fig. S5C–J). The fruit-setting rate under normal conditions was 89.2%, whereas it was substantially reduced under the low-temperature conditions: only 25% in the bagged and 7.5% in the naked berries (Fig. S5K). Time to fruit maturation extended from 40 to 80 days after fertilization (Fig. S5L). In both naked and bagged treatments, the fruit weight was <2.0 g (Fig. S5M), and the number of seeds per berry was <10 (Fig. S5H–J and N), which was a substantial difference compared to fruits developed under normal conditions.

These results suggest low temperature is deleterious to fruit development in *Physalis* and tomato; however, the Chinese lanterns protect berries from low-temperature environments and thus improve plant fitness.

### Chinese lanterns facilitate fruit dispersal

**Figure 4:**
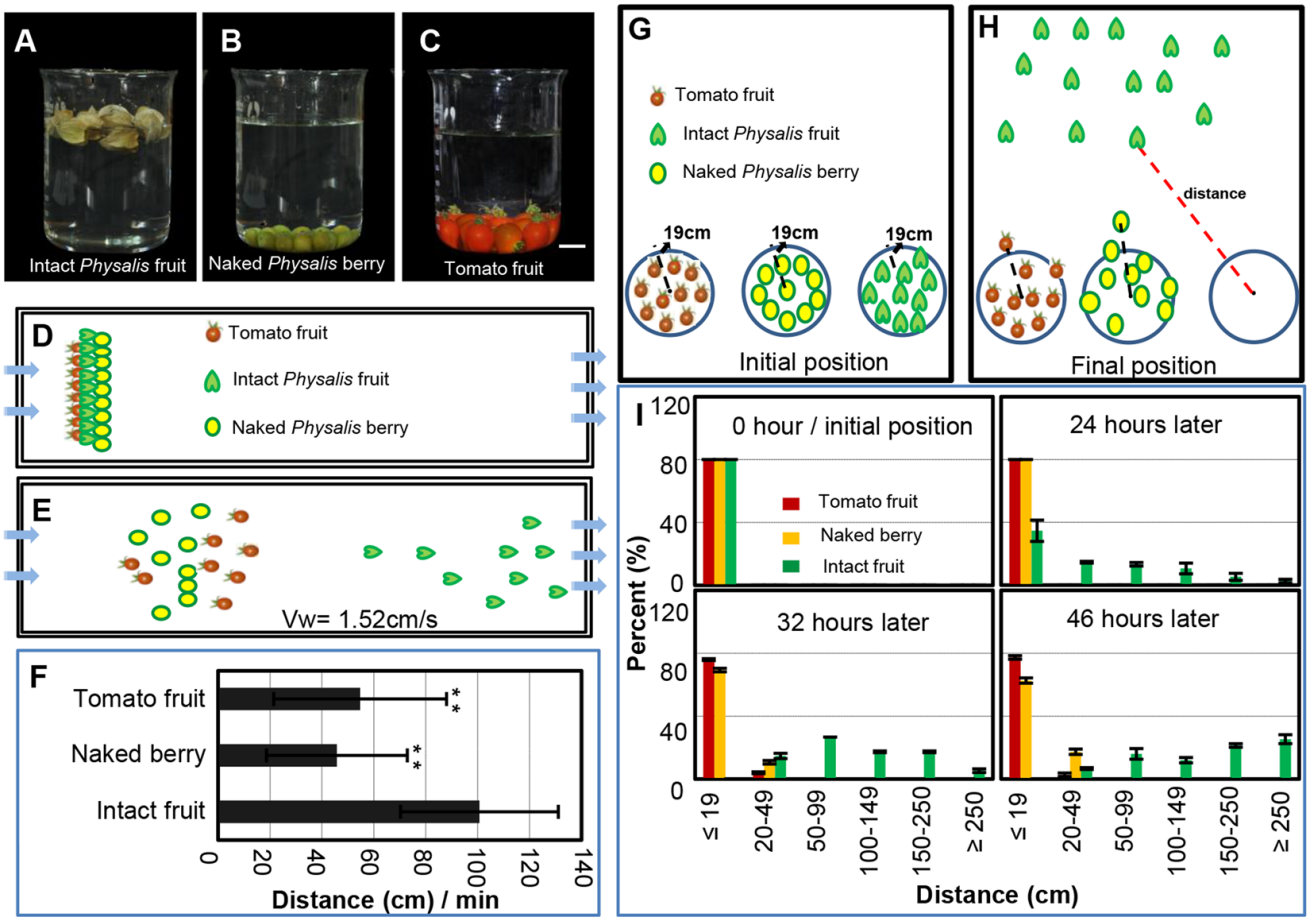
Chinese lantern facilitates fruit dispersal. (**A–C**) Water-floating ability of fruits. (**A**) The intact *Physalis floridana* fruits. (**B**) The naked *P. floridana* berries. (**C**) The *S. pimpinellifolium* fruits. Bar = 1 cm. (**D–F**) Fruit dispersal mediated by water. (**D**) Diagram explanatory of fruit dispersal in the water. Tomato berry, naked berry and berry with the Chinese lantern started at one plane as indicated. (**E**) A demonstration of fruit distribution after 1 min water flow. Arrows indicate water flow direction. (**F**) Quantification of fruit dispersal distance as indicated in 1 min. Water flow speed was 1.52 cm/s. (**G–I**) Fruit dispersal by wind. (**G**) Diagrammatic demonstration of initiation setting. The indicated fruits were put in a circle with a radius of 19 cm as indicated. (**H**) A demonstration of fruit distribution after wind treatment. Spread distance indicated by dashed lines. (**I**) Quantification of the distance of fruit dispersal as indicated by time points. The significance of differences is indicated by ^*^(*P* ≤ 0.05) and ^**^(*P* ≤ 0.01).

We next examined how the Chinese lantern might facilitate fruit dispersal after fruit maturation. First, we put *Physalis* and tomato fruits in water and found that *Physalis* fruits floated for a long period of time until water entered the lantern; in contrast, when the calyx was removed, the naked *Physalis* berries immediately sank (as the tomato berries did) (Fig. 4A–C), indicating that *Physalis* fruit dispersal would be mediated by water. We designed a water flow experiment to evaluate this. The water-control device was 5 m long and the velocity was controlled in the tank with two inlets and three outlets (Fig. 4D). Tomato fruits, naked *Physalis* berries and intact *Physalis* fruits were placed on one line (as indicated in Fig. 4D), and both tomato berries and the naked *Physalis* berries sank to the bottom whereas the intact *Physalis* fruits floated. The subjected berries had different stop points after a certain period (Fig. 4E). If water velocity was kept at about 1.5 cm/s, compared with the tomato and naked *Physalis* berries, the distance moved of the intact *Physalis* fruits in 1 min was significantly longer, floating up to 100 cm away from the start point (Fig. 4E,F). No significant difference was observed between the dispersal distances of the tomatoes and the naked *Physalis* berries (Fig. 4F). Therefore, the Chinese lantern may offer a fitness advantage by facilitating dispersal of fruits by flotation.

To investigate whether lanterns facilitate wind dispersal, we performed the following experiment on consecutive windy days. Tomato fruits, naked *Physalis* berries and the intact *Physalis* fruits were placed into three circles (indicated in Fig. 4G). In windy environments, berries/fruits could deviate from the circle (Fig. 4H). From weather forecast data, we selected three consecutive days each year (over three years) when there was a northwest wind of 20–38 km/h. In each experiment, the final distance the berry deviated from the circle center was recorded. After 24 h, compared with the original set (Fig. 4I), the tomato fruits and the naked *Physalis* berries were unmoved, whereas around 56.7% of *Physalis* fruits with a Chinese lantern were spread to different distances, and of which around 3.3% of the total were moved more than 250 cm from the center of the circle (Fig. 4I). Similar patterns were observed after 32 and 46 h; nonetheless, intact *Physalis* fruits deviated far away from the initial position and some fruits were not found (Fig. 4I). In contrast, all tomato fruits and naked *Physalis* berries mostly remained in the circle, and only a small portion of tomato fruits (5.0%) and naked *Physalis* berries (21.7%) had moved <50 cm (Fig. 4I). Therefore, *Physalis* berries containing the Chinese lantern structure could be more efficiently dispersed by wind than those that had the lantern removed.

## Discussion

Revealing the selective values of morphological novelty is essential to understand the origin of the novelty itself. Through experimental approaches, we, for the first time, investigated biological and ecological role of the Chinese lantern, a morphological novelty in *Physalis*, to look for evidence of evolutionary advantages of this morphological innovation. We found that the Chinese lantern played multiple functions during fruit development in *Physalis* and provided fitness benefits.

### Photosynthesis for fruit development

The Chinese lantern structure is derived from an accrescent calyx, collective of sepals^11^, which are ultimately modifications of leaves^21^. Thus, the calyx (sepals) might have the primary function of green leaves in producing photosynthate^22,23^. Calyx removal has an effect on the delivery of sucrose into sink organs, and leads to reduction in fruit weight in *Diospyros kaki*^24,25^. In tomato, the green calyx contributes to 2% of fruit weight^22^. In *Physalis*, through calyx removal and physiological tests, we found that the inflated green calyx also serves a photosynthetic source for fruit and seed development. The wounding effect on fruit development was not excluded; nonetheless, calyx removal at the blooming stage led to significant reductions in fruit and seed weight in the naked *Physalis* berries, indicating that photosynthesis in the inflated calyx of *Physalis* very likely contributes to fruit and seed development. The photosynthetic role of the green calyx is not surprising because this activity is a fundamental attribute of green plant tissues^26,27^. However, bagging the naked *Physalis* berry after calyx removal rescued around 10% of the weights of berries and seeds, implying that the observed weight reduction resulted from more than the photosynthetic capability of the green lanterns.

Our findings are consistent with previous observations in *Helleborus foetidus* (Ranunculaceae), which has large, persistent green sepals that contribute to fitness (enhanced seed number and size)^8^. Our data also suggest that the inflated calyx could improve plant fitness in *Physalis*. However, unlike the enlarged and flat sepals in *H. foetidus*^8^, the fruiting calyx of *Physalis* develops into a lantern with a small opening at its tips, thus forming a relatively closed microclimate or chamber. The differences we observed in fruit and seed weights between naked and bagged *Physalis* berries in these calyx-removal experiments implies that the Chinese lantern structure provides a microclimate beneficial to fruit and seed development. Moreover, the amount of chlorophyll was extremely small and the rate of photosynthesis of the Chinese lanterns was quite low, and they had a tendency to decline as the lanterns developed. Calyx removal at later stages (after pollination) had less of an effect on berry development^12^; however, the photosynthetic output of the lanterns, which revealed by removing the calyx at the blooming stage, seemed to be significant. The underlying mechanisms for this striking contrast are unclear, but it might be partially associated with microclimate effects of the Chinese lanterns (see below).

### Microclimate effects in fruit development improve fitness

Variation in the external environment affects seed number and size^28^, and thus plant fitness. Calyx-removal from floral buds suggested that the floral calyx is important to fruit development in *Physalis*^12^. In this study, seed number per berry and seed size were reduced; additionally, fruit setting, survival rate, and fruit weight were reduced among calyx-removed treatments at the blooming stage following artificial pollination. We thus predicted that the Chinese lantern structure is beneficial to fitness. We also observed that the lantern can buffer the inside temperature and humidity as the external environment changes. Under 16 °C conditions, the internal temperature and relative humidity were higher inside the Chinese lanterns, providing relatively optimal microenvironments. The crucial contributing role of Chinese lantern to fruit development and fitness, such as by controlling microclimate, was further demonstrated under the low-temperature conditions. Moderate low temperature (approximately 19 °C) disrupts pollen development and causes severe reductions in rice grain yields via reducing gibberellin^29^. To avoid the cool temperature damage at floral stages, *Physalis* seedlings at the blooming stage were transferred into 16 °C constant incubators, and artificial pollination was carried out. Under the low temperature, intact *Physalis* fruit development was significantly retarded and fruits needed twice the amount of time to mature compared with naturally developed fruits; moreover, fruit setting rate (after artificial pollination), and the weights of berries and seeds were dramatically reduced. After removing the floral calyx under low temperature, although fertilization could be done, the naked *Physalis* berries hardly ripened, and the seeds in these berries never became mature. However, the bagged berries could counter the negative effects that resulted from calyx removal under the low-temperature conditions. Furthermore, Chinese lanterns, as a natural barrier, might also protect the developing berry from animals and improve the survival of mature berries^30^ (Fig. S6). Therefore, Chinese lanterns, as a microclimate chamber, are required for fruit growth, development, and maturation, improving *Physalis* fitness. This prediction was further supported by the observations of tomatoes bagged under the low-temperature conditions, having significantly improved fitness. A similar strategy to improve fitness might occur during floral differentiation in some plants. For example, temporal floral closure forms a transient “chamber” and benefits reproduction of *Magnolia denudata*^31^. But little is currently understood about how a small change in temperature (<2 °C) or humidity (<5%) between the inside and outside of the Chinese lantern could make such a tremendous effect on berry development and fitness. However, the reproductive phase in flowering plants is often highly sensitive to external environmental alterations; for example, pollen development and fertilization may often be the most sensitive reproductive stages^29,32,33^. We, therefore, surmise that the microclimate provided by the Chinese lantern structure formed just after fertilization might mainly exert its role to improve fitness during the short time surrounding fertilization.

### Multiple dispersal strategies of fruits and seeds

A ripe fruit can be dispersed away from its mother plant by multiple mechanisms. Seed dispersal of sweet, fleshy fruits, such as berries, is performed by frugivorous animals, such as bats and birds^34–36^, whereas wind-dispersed seeds depend on high seed production, aerodynamic seed morphology, and small seed size^37–41^. Fruits may develop certain novel structures, like wings, thorns, or an explosive apparatus to facilitate seed dispersal^42–45^. Solanaceous species present remarkable fruit morphological diversity^9,14,15,46^, which contributes to a wide dispersal of fruits and seeds. Most berries, like tomato and eggplants, are dispersed by animals (including humans), while dispersal of a few could be mediated by non-organism forces^9^. The Chinese lantern, as the accessory organ of fruit, can facilitate fruit and seed dispersal. In water, tomatoes basically sink to the bottom immediately, whereas intact *Physalis* fruits usually float for several days. Moreover, intact *Physalis* fruits moved farther than the tomato berries as water flowed. Lantern removal made the naked *Physalis* berries disperse more like tomatoes in water. These observations suggested potential adaptive functions of the Chinese lantern in fruit dispersal in water. Interestingly, fossil discovery of the inflated calyces suggest that *Physalis* originated in ancient, humid, and riparian environments, such as the lakeside rainforest paleoenvironment^18^, thus suggesting the possible primary role of the Chinese lantern in floatation dispersal.

Nonetheless, the nature of the lantern- or balloon-like structure permits other strategies to fulfill long distance dispersal. Long distance dispersal of the intact *Physalis* fruits is efficiently mediated by wind; however, lantern removal disabled the capability of long distance dispersal. The mobility of tomato berries in wind was severely limited, as was the naked *Physalis* berries. Consistent with this, wind plays a role in fruit dispersal of *Przewalskia tangutica*^9^ which has a lantern-like structure, the homolog of the Chinese lantern in *Physalis*.

The function of the intact *Physalis* fruits in these abiotic conditions is attributed to the nature of the Chinese lantern structure. However, animal dispersal (human and birds) of *Physalis* fruits and seeds does exist^47,48^. Unlike *P. floridana*, mature fruits of *Physalis alkekengi* are not easy to separate from mother plants (Fig. S6A–C). In this species the Chinese lantern structure was gradually decayed by rains and winds, and then the red berry became visible for the seed disseminators (Fig. S6D). In this scenario, Chinese lanterns provide a microclimate to keep mature berry fresh till consumers capture and disperse seeds.

### The origin of the lantern trait

The Chinese lantern structure is an advantageous and adaptive structure in *Physalis*, and similar morphological innovations have been observed in various plants. Analogous to the inflated calyx in a few genera in Solanaceae^10,11,16^, the carpels in *Koelreuteria* and *Staphylea* are inflated^49,50^. The inflated floral organs in these plants are not homologous to those in *Physalis*, but they might have similar functions in fruit and seed development and dispersal, reflecting convergent evolution. Therefore, the assumption that ICS-like structure seems to be directly selected is well supported. However, revealing the developmental evolutionary genetics of the Chinese lantern suggested that the origin of the novelty is likely coupled with male fertility in *Physalis*^11,13,20,51^, thus, raising the hypothesis that the Chinese lantern structure might be also a hitchhiking byproduct, an idea needing further investigation. The plesiomorphic and ancient origin of the lantern trait^10,18^, and secondary mutations leading to the trait loss in most genera within the Solanaceae^10,16,17^ seems to support this assumption. Moreover, a few morphological novelties are believed to be evolutionary byproducts^52–55^.

## Conclusions

The persistence and accrescence of the calyx may be an advantageous trait in plants. In *Physalis*, the green fruiting calyx photosynthesizes and the Chinese lantern that develops after flowering serves protective roles by controlling microclimate. These benefits aid fruit and seed development, suggesting that the lantern structure improves fitness, although the mechanisms and pathways that the Chinese lantern impacts plant fitness need addressing. Moreover, this novel structure facilitates fruit and seed dispersal such that fruits having these ICS-like structures may have greater potential to be distributed to wider ecological niches. Additional roles of the Chinese lantern, as observed in *Physalis angulata*, are to provide a refuge for the larva of *Heliothis subflexa*^56^ and others, in which developing berries could nourish the larva (personal observations), suggesting that the novel structure, although immediately maladaptive to the plant in decreasing reproductive fitness, might play a role in maintaining a local ecological system. The origin of the lantern trait is still in debate, but the Chinese lantern, as a masterpiece of nature, clearly influences both plant fitness and ecological relationships.

## Methods

### Plant materials

*Physalis floridana* P106^11^ and *Solanum pimpinellifolium* (LA1589) were grown in an experimental field at the Institute of Botany, Chinese Academy of Sciences, under natural conditions from May to September in the years 2014, 2015, and 2016. The P106 and Micro-Tom plants for all controlled experiments were grown in plant-growth incubators either under 16 °C or 28 °C.

### Calyx area quantification

The unfolded floral and fruiting calyces were imaged, and then calyx areas were quantified using the software, NIS-Elmenys3.1. Twenty calyces at each defined developmental stage were measured.

### Determination of chlorophyll content

The leaf near to the fruit and calyces of 5 days post fertilization (DF), 20 DF and mature fruit (MFr) were collected and crushed in absolute alcohol. The mixture was thoroughly shaken and then kept in the dark for 16 h. Absorbance of the supernatant was recorded at 663 and 645 nm on a UV-visible spectrophotometer (ChemitoSpectrascan, UV 2600, Chemito Instruments Pvt. Ltd.; Mumbai, India). The chlorophyll content was estimated according to Arnon (1949)^57^.

### Photosynthetic efficiency measurement

Photon flux density response curves were measured by GFS-3000 and DUAL-PAM-100 measuring systems (Heinz Walz, Effeltrich, Germany). The net photosynthetic rate (Pn, μmol·m^−2^·s^−1^) was determined under the following eight photon flux density gradients: PS1450, PS918, PS606, PS325, PS167, PS89, PS31, and PS0. Further light response curve fitting was done using a non-rectangular hyperbola model to confirm photosynthetic characteristics^58^. Apparent quantum efficiency (AQE, mol·mol^−1^), the light compensation point (LCP, μmol·m^−2^·s^−1^) and light saturation point (LSP, μmol·m^−2^·s^−1^) difference, and the max net photosynthetic rate [Pn (max), μmol·m^−2^·s^−1^] were used to reflect capacity of light utilization and photosynthetic productivity efficiency of green tissues, while dark respiration rate (Rd, μmol·m^−2^·s^−1^) quantifies the material consumption of respiration.

### Temperature and relative humidity (RH) measurement

For the diurnal variation, both temperature and RH of inner calyx and external environment were recorded every 1 h on Aug. 15, 23, and 27 of the three cultivated years in the experimental field. Five lanterns for each developmental stage [5 DF, 20 DF and MFr that represent the developing fruits 5 and 20 days after fertilization (DF) and mature fruits (MFr)] from three plants were measured for three days in each experiment per year. For temperature analyses, the *Physalis* plants were placed into an incubator set at 28 °C. The temperature and RH inside and outside of each Chinese lantern (20 DF) were simultaneously measured as the temperature was dropped (in 3 °C decrements). We also measured these parameters at a constant temperature of 16 °C. Ten lanterns for each developmental stage from three plants were treated in each experiment. Temperature and relative humidity were recorded using a dew point measuring instrument (Testo 635, TestoSE & Co. KGaA, Lenzkirch, Black Forest, Germany).

### Calyx-removal analysis

Calyces were completely removed from *P. floridana* at the blooming stage, and then flowers were artificially pollinated; the resultant fruits were designated as the naked *Physalis* berries. In parallel, some naked berries were covered using waterproof and non-transparent paper bag (7.5 cm × 10.5 cm) (Laiyang paper Industry, Yantai, China). These berries as well as the intact fruits from artificial pollination were labeled simultaneously. Fifty samples were included in one treatment, and each treatment was replicated at least three times. The fruit setting or survival rate, wet and dry fruit weight, seed weight and seed number per berry were measured. The fruit maturation time was defined as the time from pollination to maturation. The experiments were performed in an experimental field and in 16 °C incubators.

### Low temperature treatment

*Physalis* (P106) and tomato (Micro-Tom) seedlings at the blooming stage were transferred into 16 °C constant incubators. For *Physalis*, the experiment was done as in the section “Calyx-removal analysis”. Pollen activity was detected using the iodine-potassium iodide (I_2_–KI) staining. For tomato, 40 blooming flowers were bagged with waterproof paper (7.5 cm × 10.5 cm), and 40 flowers were kept naked. We recorded the number of growing days from flowers to mature fruits, the rate of fruit set, fruit weight, and number of seeds per berry. Tomato plants growing in normal conditions were used as control.

### Fruit dehydration

Naked berries and intact fruits of *Physalis* were placed as two groups on an experimental bench at 27 °C for one month. Fruit weight was measured weekly. Thirty fruit samples were measured in each group. One typical fruit image for each group was photographed.

### Fruit dispersal analyses

To investigate the fruit-dispersal capability in water, a rectangular iron tank (length × width × depth = 5.0 m × 0.3 m × 0.3 m) with two inlets and three outlets was made as a water flow device (a schematic map is presented in the Results section; Fig. 4D,E). Before the experiments, water was filled in the tank. The naked berries and intact fruits of *Physalis* were placed into water at one vertical plane, and both the inlets and the outlets were opened simultaneously. The flow distances of the subjected berries/fruits were measured after 1 min. Ten fruits or berries were used in each experiment, and the experiment was repeated 20 times. To test dispersal ability in wind, we chose three consecutive days during which there was a northwest wind of 20–38 km/h. The naked berries and intact fruits of *Physalis* were placed in a circle with 19-cm radius (for details, see the Results section; Fig. 4G,H). The distances from which the fruits deviated from the center of the circle were recorded after 24, 32 and 46 h. We took 30 samples of each treatment fruit, and the experiments were performed three times. The tomatoes from *S. pimpinellifolium* were included as controls in all these experiments.

### Statistical analysis

The recorded data were analyzed in EXCEL, and the significance between treatments was evaluated on the two-tailed Student’s *t*-tests by using SPSS 24.0 (IBM Corp, New York, NY, USA).

## Electronic supplementary material

Electronic supplementary material

## Data Availability

All data and materials involved in this study are included in this published article and its supplementary file.
