# Supplementary material for: Chinese lantern in Physalis is an advantageous morphological novelty and improves plant fitness
Source: Sci Rep. 2019 Jan 24;9:596. doi: 10.1038/s41598-018-36436-7 (PMC6345875; doi:10.1038/s41598-018-36436-7)
Supplement: Supplementary file 1 — Electronic supplementary material [file 41598_2018_36436_MOESM1_ESM.pdf]

Supplementary Information

Chinese lantern in *Physalis* is an advantageous morphological novelty and improves plant fitness

Jing Li[1,2], Chunjing Song[1,2] and Chaoying He[1,2,*]

1 State Key Laboratory of Systematic and Evolutionary Botany, Institute of Botany, Chinese Academy of Sciences, Nanxincun 20, Xiangshan, 100093 Beijing, China;

2 University of Chinese Academy of Sciences, Yuquan Road 19, 100049 Beijing, China.

* For correspondence: Tel: +86-10-62836085; Fax: +86-10-62590843; E-mail: chaoying@ibcas.ac.cn

Supplementary Information contains: Supplementary Figures S1-S6

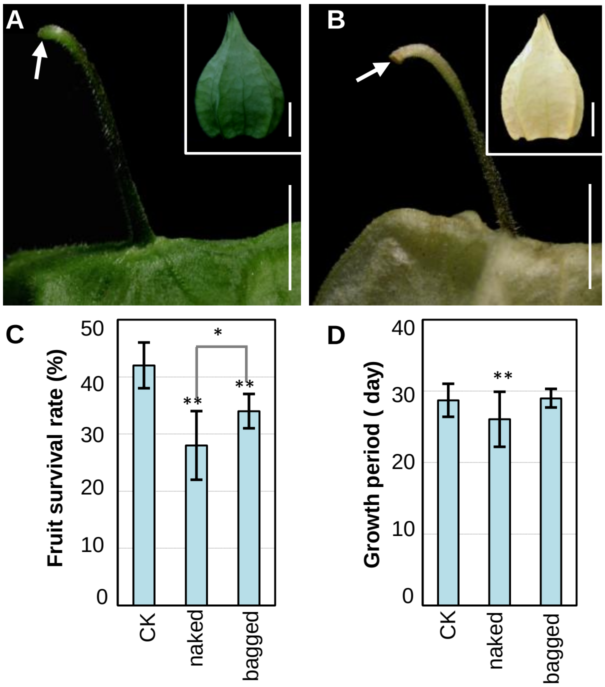

Figure S1 Fruit maturation in *Physalis floridana*.
(A) Morphology of immature fruit. **(B)** Morphology of mature fruit. Carpopodium apex of immature (**A**) and mature (**B**) fruit is indicated by the arrows. Bar = 1 cm. **(C)** Fruit survival rates under natural conditions. **(D)** Effect of microclimate on fruit maturation. CK, the intact *Physalis* fruits; naked, the naked *Physalis* berries; bagged, the bagged *Physalis* berries after removing the calyx. ** indicates the significance of differences at $P \leq 0.01$.

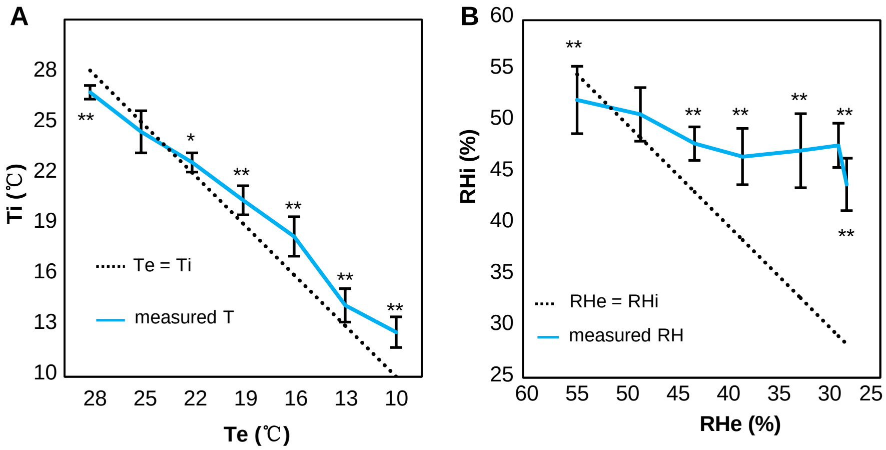

Figure S2. Chinese lantern is a microclimate-keeper in *P. floridana*.
(**A**) Ti variation of the Chinese lanterns as Te dropped (in 3 ℃ decrements). Ti and Te indicate internal and external temperature of the lanterns, respectively.
(**B**) RHi variation of the Chinese lanterns. RHi and RHe indicate internal and external RH of Chinese lanterns, respectively. The significance of difference is indicated by * ($P \leq 0.05$) and ** ($P \leq 0.01$).

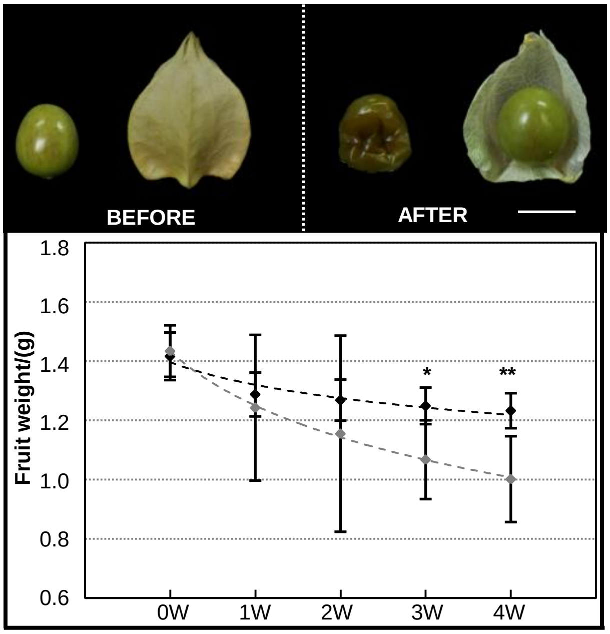

Figure S3 Chinese lantern slows berry dehydration. Images show the naked berry and the intact fruit under different conditions as indicated as BEFORE and AFTER that respectively indicate the initial state of the fruits and the fruit state of 4 weeks exposure to 27 ℃. Part of the calyx from an intact fruit was removed to show the berry inside. Bar = 1 cm. The graph shows quantification of the variation in fruit weights. The black and grey dashed lines indicate the berry weight of the intact fruits and the naked berries, respectively. Fruits were weighed weekly (W). Thirty samples for each group were included. The significance of differences is indicated by * ($P \leq 0.05$) and ** ($P \leq 0.01$).

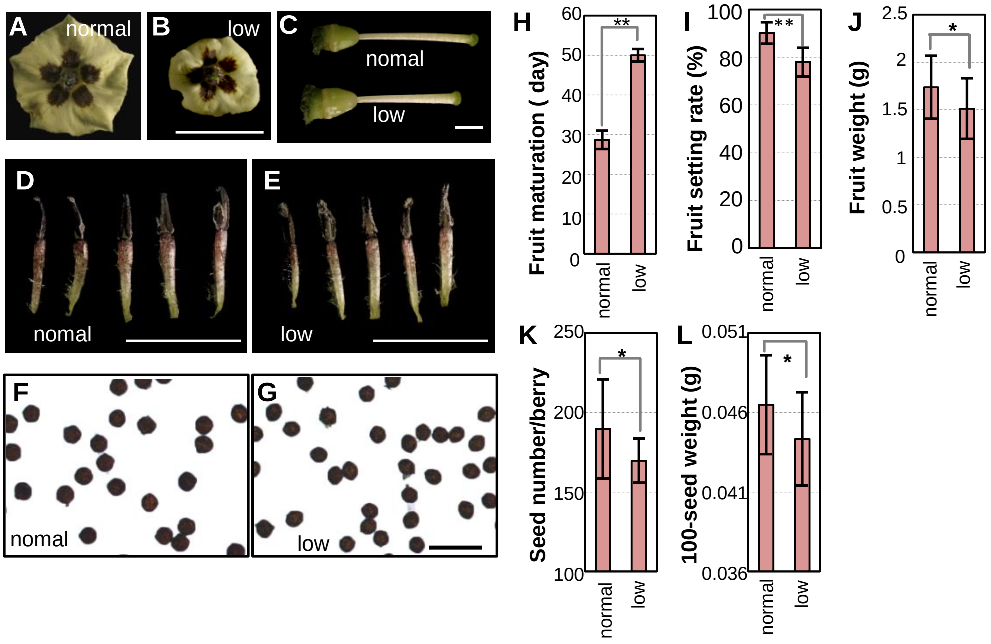

Figure S4 Low temperature (16 ℃) reduces fitness of *Physalis*.
(**A)** A flower developed under normal conditions. **(B)** A flower developed under low-temperature conditions. Bar = 1 cm. **(C)** A comparison of carpel that developed under both normal and low temperature conditions. Bar = 1 mm. **(D)** The anthers from a flower developed under normal conditions. **(E**) The anthers from a flower developed under the low temperature conditions. Bar = 5 mm. (**F-G**) Pollen maturation with I_2-KI stain from normal and low temperature conditions. (**H–K**) Quantification of fruit maturation (**H**), fruit setting rate **(I)** fruit weight (**J**), seed number per berry (**K**) and 100-seed weight (**L**) of plants from normal and low temperature conditions. The plants were transferred to 16 ℃ at the blooming stage.

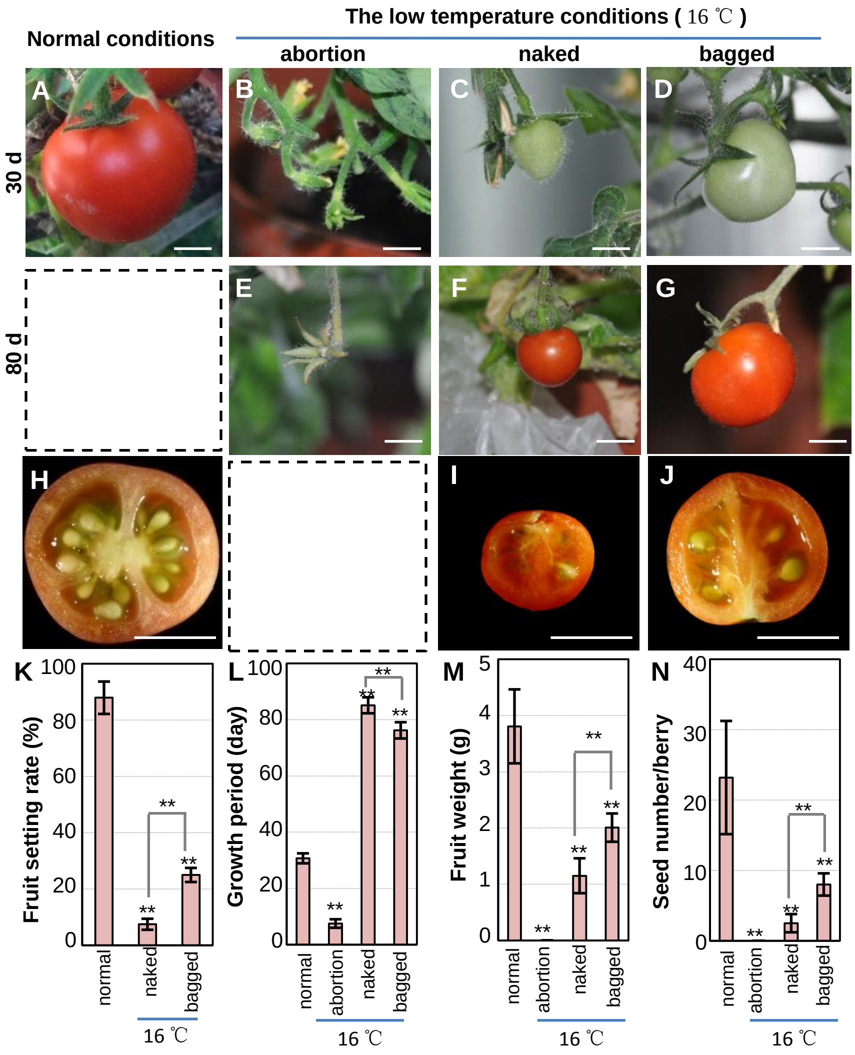

Figure S5 Bagging partially rescues the fitness reduction in tomato caused by low temperature.

(A–D) The tomato fruits 30 d after fertilization at the indicated conditions. **(A)** A mature tomato. **(B)** The aborted fruits. (**C**) A naked developed fruit. (**D**) A bagged developed tomato. (**E–G**) The tomato fruits 80 days after fertilization at the indicated conditions. **(E)** An aborted tomato. (**F**) A mature naked tomato. **(G)** A mature bagged fruit. (**H–J**) Sections of the mature fruits at the indicated conditions to show the seeds. **(H)** A mature tomato. **(I)** A mature naked tomato. **(J)**. A mature bagged tomato. The dashed box indicates the absence of the fruit state at the indicated conditions. Bar = 5 mm. (**K–N**) quantification of the traits under the indicated conditions. **(K)** Fruit setting rate. **(L)** Fruit maturation time. **(M)** Fruit weight. (**N**) Seed number per berry. The Micro-Tom was used in this case. The significance of difference is indicated by * ($P \leq 0.05$) and ** ($P \leq 0.01$).

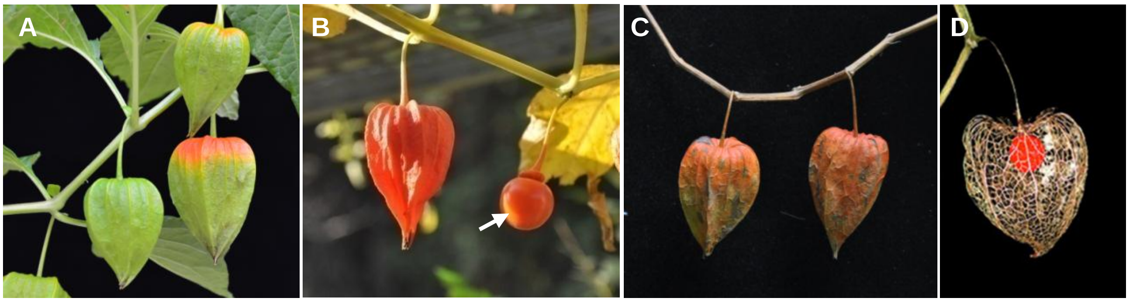

Figure S6 Chinese lanterns of *Physalis alkekengi*.
(**A**) Immature fruits. (**B**) Mature fruits on the living plant. The fruiting calyx of a fruit is removed to show the berry indicated by an arrow. (**C**) Mature fruits on a dead plant. (**D**) A mature fruit. A calyx destroyed by rain; frugivores such as birds could then see and remove the berries.
